# Overexpression of *Artemisia annua* Cinnamyl Alcohol Dehydrogenase Increases Lignin and Coumarin and Reduces Artemisinin and Other Sesquiterpenes

**DOI:** 10.3389/fpls.2018.00828

**Published:** 2018-06-19

**Authors:** Dongming Ma, Chong Xu, Fatima Alejos-Gonzalez, Hong Wang, Jinfen Yang, Rika Judd, De-Yu Xie

**Affiliations:** ^1^Research Center of Chinese Herbal Resource Science and Engineering, Guangzhou University of Chinese Medicine, Guangzhou, China; ^2^Department of Plant & Microbial Biology, North Carolina State University, Raleigh, NC, United States; ^3^Graduate University of Chinese Academy of Sciences, Beijing, China

**Keywords:** *Artemisia annua*, artemisinin, arteannuin B, cinnamyl alcohol dehydrogenases, coumarin, lignin, sesquiterpenes

## Abstract

*Artemisia annua* is the only medicinal crop that produces artemisinin for malarial treatment. Herein, we describe the cloning of a cinnamyl alcohol dehydrogenase (AaCAD) from an inbred self-pollinating (SP) *A. annua* cultivar and its effects on lignin and artemisinin production. A recombinant AaCAD was purified via heterogeneous expression. Enzyme assays showed that the recombinant AaCAD converted p-coumaryl, coniferyl, and sinapyl aldehydes to their corresponding alcohols, which are key intermediates involved in the biosynthesis of lignin. Km, Vmax, and Vmax/Km values were calculated for all three substrates. To characterize its function *in planta*, *AaCAD* was overexpressed in SP plants. Quantification using acetyl bromide (AcBr) showed significantly higher lignin contents in transgenics compared with wild-type (WT) plants. Moreover, GC-MS-based profiling revealed a significant increase in coumarin contents in transgenic plants. By contrast, HPLC-MS analysis showed significantly reduced artemisinin contents in transgenics compared with WT plants. Furthermore, GC-MS analysis revealed a decrease in the contents of arteannuin B and six other sesquiterpenes in transgenic plants. Confocal microscopy analysis showed the cytosolic localization of AaCAD. These data demonstrate that AaCAD plays a dual pathway function in the cytosol, in which it positively enhances lignin formation but negatively controls artemisinin formation. Based on these data, crosstalk between these two pathways mediated by AaCAD catalysis is discussed to understand the metabolic control of artemisinin biosynthesis in plants for high production.

## Introduction

**FIGURE 1:**
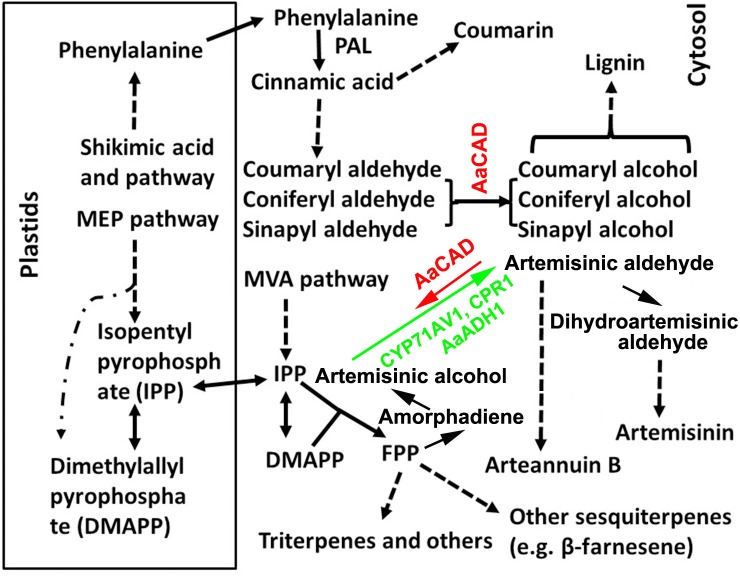
A simplified scheme showing the cellular compartmentation associated with the biosynthesis of lignin, coumarin, artemisinin, and other metabolites. Pathway abbreviations: MEP, 2-C-methyl-D-erythritol 4-phosphate and MVA, mevalonate pathway; enzyme abbreviations; ADS, amorphadiene synthase; AaADH1, *Artemisia annua* alcohol dehydrogenase; AaCAD, *Artemisia annua* cinnamyl alcohol dehydrogenase; CPR1, cytochrome P450 reductase 1; CYP71AV1, cytochrome P450 mono-oxygenase; and PAL, phenylalanine ammonia-lyase. Dashed arrows and lines denote multiple steps. Solid arrows denote one step. The short arrow for AaCAD in the artemisinin pathway denotes a partial reverse. The long arrow for CYP71AV1 and CPR1 denotes the main direction toward artemisinin.

Artemisinin-based combination therapy (ACT) is the first-line treatment for malaria (WHO, 2006, 2013; Maude et al., 2009). *Artemisia annua* L. (sweet wormwood), an effective antimalarial plant, is the only natural resource to produce artemisinin, an endoperoxide sesquiterpene lactone. Due to the global demand for ACT, understanding artemisinin biosynthesis in this medicinal plant is critical for metabolic engineering purposes for high production of this sesquiterpene. To date, numerous studies have molecularly and biochemically characterized the main steps of the artemisinin biosynthetic pathway, starting with amorpha-4,11-diene (amorphadiene) localized in the cytosol of glandular trichomes (**Figure 1**) (Bouwmeester et al., 1999; Teoh et al., 2006; Covello et al., 2007; Zhang et al., 2008; Czechowski et al., 2016). A recent review summarized enzyme assay, synthetic biology, and multiple transgenic studies that solidly demonstrate the first step catalyzed by amorpha-4, 11-diene synthase (ADS) s (Xie et al., 2016). The second step is catalyzed by a cytochrome P450 mono-oxygenase (CYP71AV1) coupled with a cytochrome P450 reductase 1 (CPR1). These two enzymes were originally demonstrated to convert amorpha-4, 11-diene to artemisinic alcohol and then to artemisinic aldehyde (Ro et al., 2006;Teoh et al., 2006). Recently, a new gene encoding an *A. annua* alcohol dehydrogenase 1 (AaADH1) was cloned from glandular trichomes of *A. annua* and demonstrated to catalyze the conversion of artemisinic alcohol to artemisinic aldehyde (Paddon et al., 2013). This discovery has greatly enhanced our understanding of the second step. A double-bond reductase (DBR) and an aldehyde dehydrogenase (ALDH) subsequently convert artemisinic aldehyde to dihydroartemisinic aldehyde and then dihydroartemisinic acid (DHAA) (Xie et al., 2016). The spontaneous oxidation of DHAA finally produces artemisinin. In addition, arteannuin B is derived from artemisinic aldehyde (**Figure 1**). Recently, a *cyp71av1* mutant of *A. annua* was generated to provide fundamental genetic evidence demonstrating the essential role of CYP71AV1 in controlling the artemisinin biosynthetic pathway *in planta* (Czechowski et al., 2016). This mutant further provided solid evidence to demonstrate the formation of artemisinin through spontaneous oxidation. In addition to research conducted in plants, fundamental successes in synthetic biology have further demonstrated the steps catalyzed by ADS, CYP71AV1, CPR1, and AaADH1 (Ro et al., 2006; Paddon et al., 2013; Turconi et al., 2014). Although these previous achievements have demonstrated fundamental promising methods to improve the artemisinin supply, global production from current sweet wormwood crops still lacks stability and is unable to meet the increase in medicinal demands (Paddon et al., 2013; Ma et al., 2017a). Therefore, continuous research efforts are urgently necessary to elucidate the regulatory mechanisms of artemisinin biosynthesis to improve the global yield.

Cinnamyl alcohol dehydrogenase (CAD) is categorized in a group of short-chain oxidoreductases in the family of nicotinamide adenine dinucleotide phosphate (reduced form)-dependent enzymes. It has been appropriately characterized to catalyze the conversion of phenylpropenyl aldehydes to alcohols in the late steps of lignin biosynthesis (**Figure 1**) (Sarni et al., 1984; Somers et al., 1995; Baucher et al., 1999; Chabannes et al., 2001). A large number of studies have succeeded in manipulating *CAD* expression in potent biotechnological efforts to reduce lignin in trees and crops for different economic applications (Baucher et al., 1999; MacKay et al., 1999; Chabannes et al., 2001; Fu et al., 2011; d’Yvoire et al., 2013; Trabucco et al., 2013; Anderson et al., 2015; Ozparpucu et al., 2017). The main successes associated with using *CAD* downregulation include improved digestibility of forage crops, reduction of lignin in trees for pulping and biofuel, and different renewable plants for biofuel feedstock (Lapierre et al., 2000; Fu et al., 2011; Wang Y.H. et al., 2015; Ozparpucu et al., 2017; Ponniah et al., 2017). For example, downregulation of *CAD* in alfalfa has been shown to lead to a decrease in lignin and improved digestibility of this forage crop (Baucher et al., 1999). Suppression of *CAD* in rice has demonstrated the potential to facilitate cellulose production for biofuel feedstock (Ponniah et al., 2017). A downregulation of *CAD* has also been reported to improve saccharification in switchgrass for biofuel conversion (Fu et al., 2011).

In our previous study, we reported the cloning of a *CAD* homolog from glandular trichomes of an heterozygous (cross-pollination) *A. annua* cultivar and *in vitro* enzyme assays to show that it used cinnamyl aldehyde, coniferyl aldehyde, sinapyl aldehyde, and artemisinic aldehyde as substrates (Li et al., 2012). To date, whether this CAD can affect lignin and other metabolic pathways in *A. annua* remains to be investigated. Herein, we report the cloning of a new *CAD* homolog from a novel self-pollinating (SP) *A. annua* and characterize its enzyme kinetics and overexpression in plants. Phytochemical analysis, GC-MS-based metabolic profiling, and LC-MS analysis were conducted to characterize phenylpropanoid and terpenoid metabolism in transgenic plants. The resulting data showed that not only was this new AaCAD involved in lignin biosynthesis, but it was also associated with coumarin formation. By contrast, artemisinin and other sesquiterpenes contents were significantly decreased in transgenic plants. These data show that AaCAD can play a dual function by establishing a crosstalk between two distinct cytosolic pathways, the phenylpropanoid and sesquiterpene pathways. Although overexpression of *AaCAD* leads to a reduction of artemisinin contents, this research is very instructional for future metabolic engineering designs to improve artemisinin production in *A. annua*.

## Materials and Methods

### Plant Materials and Growth Conditions

The progeny of a self-pollinating (SP) *A. annua* variety were grown in the phytotron for seeds as described previously (Alejos-Gonzalez et al., 2011). The photoperiod and temperature in the phytotron was 16/8 h (light/dark) and 25/22°C (day and night). Seedlings of the F3 progeny were used for gene cloning, genetic transformation, and metabolite analysis.

### Cloning of *AaCAD* cDNA

DNA-free total RNA was isolated from 2-month-old seedlings of SP *A. annua* using the RNeasy Plant Mini Kit (Qiagen, United States) as described previously (Ma et al., 2015). The first-strand cDNA was synthesized with 1.0 μg of total RNA and Powerscript reverse transcriptase (Clontech). The resulting cDNA was used as template to clone *AaCAD*. A pair of primers consisting of CAD-F (5′- ATG GGA AGC ATG AAA GAA GAA AG-3′) and CAD-R (5′-ATT TGT TGT TTC CTC TTC CAA A-3′) was designed for RT-PCR, which was carried out to obtain the open reading frame (ORF) fragment of *AaCAD*. The PCR product was further sequenced to analyze its nucleotides. The resulting ORF was deduced to determine the amino acid sequence, which was aligned with a reported CAD sequence obtained from GenBank. The sequence alignment was completed using an online Cluster Omega program^1^.

### Heterogeneous Expression of Recombinant AaCAD

The ORF of *AaCAD* containing its stop codon TAA was cloned into the pENTR/D-TOPO vector (Gateway, Invitrogen) to obtain a recombinant pENTR-AaCAD plasmid. The LR Clonase II enzyme mix (Invitrogen) was used to digest the pENTR-AaCAD plasmid and the destination vector pDEST17 (6xHis tag). As a result, the *AaCAD* ORF was cloned into pDEST17 to obtain a new pDEST17-AaCAD plasmid for protein expression. All cloning steps followed the manufacturer’s protocol. The pDEST17-AaCAD plasmid was further introduced into competent *E. coli* strain BL21 cells to induce recombinant protein.

For protein induction, a single colony was selected and then cultured in 200 ml liquid LB medium supplied with 50 mg/L ampicillin at 37°C in 500-ml E-flasks. When the OD value of the suspension culture was approximately 0.6 at 600 nm, 0.1% L-arabinose and 0.1 mM IPTG were added the flask. The suspension culture was continuously incubated for an additional 20 h at 16°C. As described in our recent report (Ma et al., 2017a), the recombinant AaCAD was purified using Ni-NTA Superflow Columns (Qiagen, 1.5 ml) according to the manufacturer’s protocol. The resulting purified recombinant AaCAD was loaded onto PD-10 columns (Amersham Pharmacia Biotech, now GE Healthcare Life Sciences, http://www.gelifesciences.com) to remove salts. Sodium dodecyl sulfate polyacrylamide gel electrophoresis (SDS-PAGE) was performed to examine the purification of the recombinant AaCAD. The resulting desalted protein was used for the enzyme assay immediately or stored in a -20°C freezer for late use as described below.

### Enzyme Assay and Kinetics Analysis

Three lignin substrates, coniferyl aldehyde, p-coumaryl aldehyde, and sinapyl aldehyde (Sigma-Aldrich), were used to examine the catalytic activity of the recombinant AaCAD. Three substrates were dissolved in methanol. Enzymatic reactions were carried out in a 200-μl volume composed of 0.1 mM substrate, 50 mM Tris–HCl (pH 7.5), 0.5 mM NADPH, 2.0 mM dithiothreitol, and 1.3 μg purified recombinant AaCAD. All reactions were initiated by addition of AaCAD to the reaction mixture at 30°C. After 30 min, all reactions were stopped by addition of 15 μl of glacial acetic acid, followed by centrifugation at 10,000 ×*g* for 10 min. The resulting supernatant was pipetted into a 200-μl glass insert, which was further placed into a 2-ml glass vial for high-performance liquid chromatograph (HPLC) analysis. All experiments were repeated three times, each with three replicates.

HPLC analysis was performed on a Waters 2695 instrument. Metabolites were separated on an Agilent ZORBAX Eclipe XDB-C18 column (4.6 × 150 mm, 5 μm). Two HPLC grade solvents, methanol (solvent A) and water (solvent B), were used as the mobile phase. A gradient solvent program, which was composed of A:B from 5:95 to 95:5 from 0 to 40 min, was developed to elute the metabolites. The flow rate was 1.0 ml/min, and the injection volume was 20 μl. The wavelengths for detection of the metabolites were 260 nm and 340 nm.

To characterize the kinetics of the recombinant AaCAD, eight concentrations (6, 8, 10, 12, 14, 20, and 30 μM) of each substrate were tested. Except for the different concentrations of the substrates used, other components in the reaction mixture were the same as described above. All reactions were performed in 200 μl in 96-well microplates. Measurements of enzymatic products were performed using an Epoch Microplate reader (BioTek instruments Inc., United States). All reactions were initiated by the addition of enzyme and then maintained at 30°C for 10 min. All tubes were placed on a microplate for 40 min of declination recorded at 340 nm, which is classically used to quantify aldehyde and NADPH because they have maximum absorbance values at this wavelength (Goffner et al., 1992; Ma, 2010; Saathoff et al., 2011; Rong et al., 2016). This method uses extinction coefficients (14.7 × 10^-3^ to 19.45 × 10^-3^ M^-1^ cm^-1^) for both aldehyde and NADPH to calculate the relative contribution of each to the 340-nm signal (Hawkins and Boudet, 1994; Saathoff et al., 2011), which allows the elimination of potential spectrophotometric interference. We used this method to record absorbance values at one-min intervals for each tube (reaction). Each reaction was continuously recorded 40 times to obtain 40 values. The resulting data were analyzed using GraphPad Prism 6 software to determine the Km, Vmax, and Kcat values. All experiments were performed three times, each with three replicates.

### Development of Binary Vector, Genetic Transformation of *A. annua*, and Genotyping

The *AaCAD* ORF, including its stop codon, was cloned into the pENTR/D-TOPO vector (Gateway, Invitrogen) to obtain a new recombinant pENTR-AaCAD plasmid, which was introduced into competent *E. coli* DH5α cells. The destination vector used for the development of binary vectors was PMDC-84 (Xi et al., 2016). The pENTR-AaCAD and PMDC-84 plasmids were digested and interchanged using the LR Clonase II enzyme mixture (Invitrogen) following the manufacturer’s protocol. This cloning step resulted in a binary vector, namely, PMDC-84-AaCAD, in its T-DNA cassette of which *AaCAD* was cloned at the immediate downstream of a 2× 35S promoter, and a hygromycin gene was used for selection (**Figure 3A**). This binary vector was introduced into competent *A. tumefaciens* strain LBA4404 cells, from which one positive colony was selected for genetic transformation of *A. annua*, as described previously (Ma et al., 2015). Multiple transgenic shoots were generated on the hygromycin selection medium and rooted to obtain plantlets. More than 10 plantlets were planted in pot soil and placed in the phytotron for continuous growth to flower and then seed production. In addition, transgenic plants for other genes reported previously (Ma et al., 2015) were used as vector controls in this study. We further used a Leica MZ FLIII fluorescence stereomicroscope to examine trichomes including glandular and other shaped trichomes on the surfaces of leaves and stems of transgenic vs. wild-type plants.

Genomic DNA was extracted from leaf tissues of transformed plants using the DNeasy Plant Mini Kit (Qiagen, United States). A total of 50 ng of genomic DNA was used as template for PCR using a pair of primers consisting of a forward primer (5′-TCT AGA ACT AGT TAA TTA AGA AT-3′) designed based on the 35S promoter and a reverse primer (5′-ATT TGT TGT TTC CTC TTC CAA A-3′) designed based on *AaCAD*. The thermal program for PCR was composed of 94°C for 5 min, followed by 35 cycles at 94°C for 1 min, annealing for 1 min at 5°C, and extension of 1 min at 72°C, and a final extension at 72°C for 5 min. All PCR experiments were performed with three biological replicates, each repeated at least three times.

### Subcellular Localization Analysis

Subcellular localization of AaCAD was carried out as described in our recent reports (Ma et al., 2017a,b). In brief, a gateway technique was used to insert the *AaCAD* ORF without its stop codon into the pENTR/D-TOPO vector (Gateway, Invitrogen, United States) following the manufacturer’s protocol. This cloning generated a new recombinant plasmid, namely, pENTR/D-TOPO-CAD. The destination vector used was pSITEII-N1-enhanced green fluorescent protein (EGFP) (with the EGFP epitopic tag in the C-terminus). Then, *AaCAD* in the pENTR/D-TOPO-CAD vector was cloned into pSITEII-N1 by LR reactions following the manufacturer’s protocol. This cloning step generated a new pSITEII-N1-CAD/EGFP plasmid, in which *AaCAD* (without its stop codon) was directly ligated at the 5′-end of *EGFP*. The new plasmid was then introduced into *Agrobacterium tumefaciens* strain GV3101. A positive colony was selected and then activated for leaf agroinfiltration of *N. benthamiana* to transiently analyze protein expression. After 30 h of infection, leaf tissues were examined using a confocal microscope (Carl Zeiss). GFP fluorescence was excited at 488 nm and observed between 495 and 550 nm according to a method reported previously (Wang G.F. et al., 2015). All analyses were performed with three groups of biological replicates, each with at least three replicates.

### Reverse Transcription-Polymerase Chain Reaction and Western Blot Analysis

Total RNA was isolated from the young leaves of transgenic candidates and WT plants using an RNeasy Plant Mini Kit (Qiagen, CA, United States). Samples were then treated with DNase to obtain DNA-free RNA. The first-strand cDNA was synthesized using 1.0 μg of DNA-free RNA and Powerscript reverse transcriptase (RT, Clontech, United States). One microliter of cDNA was used as template for PCR using Taq polymerase (Promega, United States). The steps of these three types of experiments followed the manufacturers’ protocols, respectively. Reverse transcription-polymerase chain reaction (RT-PCR) was carried out for *AaCAD* transgenic and WT plants. The housekeeping gene β-actin was used as a reference control. The gene-specific primer pair for PCR was 5-ATGGGAAGCATGAAAGAAGAAAG-3 (forward primer) and 5′-ATTTGTTGTTTCCTCTTCCAAA-3′ (reverse primer). The thermal program was as described above for genotyping. RT-PCR experiments were performed with three groups of biological replicates, each with three replicates.

A polypeptide consisting of MGSMKEERKITGWAC selected from the AaCAD amino acid sequence was used for antibody development. This peptide was synthesized and then used to develop a polyclonal antibody in rabbit at Genscript Company (NJ 08854, United States) for western blot analysis. Western blot was performed as described in our recent reports (Ma et al., 2017a,b). In brief, total proteins were extracted from leaves of transgenic vs. wild-type plants and separated by SDS-PAGE. Separated proteins were transferred to a nitrocellulose membrane and then probed with anti-AaCAD antibody. An anti-rabbit IgG HPR conjugate was used as a second antibody (Promega). An enhanced chemiluminescence system (Thermo Scientific, IL, United States) was used to detect the hybridization signal for immunoblot analysis. Western blot analysis was repeated three times.

### Lignin Measurement

The lignin content was measured using the acetyl bromide (AcBr) method (Fukushima and Kerley, 2011; Moreira-Vilar et al., 2014). Small revisions were developed to obtain the protein-free cell wall fraction. All dried stems and branches of each plant were ground into powder and filtered using a sieve (120 μm). One gram of fine powder was suspended in 20 ml sodium phosphate buffer (0.1 M, pH 7.2) in a 50-ml capped polyethylene tube at 37°C for 30 min, followed by centrifugation at 4000 rpm for 10 min. The remaining pellet was suspended in 10 ml 70% ethanol, placed in an 80°C water bath for 5 min, and then centrifuged for 10 min to remove the supernatant. This step was repeated five times. The remaining pellet in the tube was suspended in 10 ml acetone at room temperature for 5 min and centrifuged for 5 min to remove the supernatant. These treatments obtained protein-free cell wall fraction. The pellet was completely dried in a 37°C oven until there was no weight change. Five milligrams of the dry residue sample was dissolved in 2.5 ml of acetyl bromide:acetic acid (1:3, v/v) solution in a glass tube. This treatment was maintained 24 h at room temperature. The mixture was completely transferred to a 10-ml volumetric flask, followed by addition of 0.35 ml of 0.5 M hydroxylamine hydrochloride. Glacial acetic acid was added to the volumetric flask to 10 ml. The flask was gently shaken in the upside-down direction to thoroughly mix the sample. The absorbance of the resulting mixture was recorded at 280 nm using an ultra-visible spectrometer. An extinction coefficient of 23.077 g^-1^ l cm^-1^ was used to calculate the AcBr concentration. The resulting data were used to calculate the contents of lignin extracted from the samples. Three biological replicates were analyzed for each plant. Each biological replicate was repeated three times.

### Extraction of Non-polar Metabolites and Gas Chromatograph-Mass Spectrometry Analysis

A protocol was developed to extract and profile nonpolar metabolites from tissues of *A. annua* (Ma et al., 2015). In brief, hexane was used to extract nonpolar metabolites from the leaves of 8–12 nodes of two-month-old plants grown in the phytotron (**Figure 3B**), and gas chromatograph-mass spectrometry (GC-MS) was performed using a gas chromatograph 6890 coupled with 5975C MSD (Agilent Technologies, United States). A RTX-5 capillary column (30 m × 0.25 mm × 0.25 μm) was used to separate the metabolites. Splitless mode was used in the inlet. The injection temperature was set at 250°C. The temperature was initially set at 60°C, then ramped to 260°C at a constant rate of 10°C/min, and held at 260°C for 25 min. Pure helium was used as the carrier gas, with a flow rate of 1 ml/min. A positive electron impact ion source (70 eV) was used to ionize compounds, and mass fragments were scanned in the range from 40 to 800 (*m*/*z*), with 4 min of solvent delay. Three biological replicates were analyzed for each genotypic plant. Each biological replicate was repeated three times.

### High-Performance Liquid Chromatography-Mass Spectrometry Analysis of Artemisinin

Leaf samples used for nonpolar metabolite analysis were also used for artemisinin measurements. Extraction of artemisinin and high-performance liquid chromatography-mass spectrometry (HPLC-MS) analysis were performed using a 2010 eV LC/UV/ESI/MS instrument (Shimadzu) following our protocols reported previously (Alejos-Gonzalez et al., 2011, 2013). Three biological replicates were analyzed for each genotypic plant, with each biological replicate repeated three times.

### Statistical Analyses

Experimental data were analyzed by one-way ANOVA. Subsequent multiple comparisons were performed using Duncan’s multiple range test. All statistical analyses were performed using IBM SPSS Statistics Professional Edition, and the statistical significance was set at *P* < 0.01.

## Results

### Kinetics of Recombinant AaCAD for Three Lignin Substrates

According to a *CAD* homolog sequence (ACB54931) that was cloned from a cross-pollinating cultivar (Li et al., 2012) and curated in the GenBank at NCBI database, we used RT-PCR to clone an *AaCAD* open reading frame (ORF) (GenBank accession ID#: MH017050) from the leaf tissues of self-pollinating (SP) *A. annua*. The full-length ORF is composed of 1086 base pairs of nucleotides that are deduced to encode 362 amino acids. An alignment between the deduced amino acid sequence and the homologous ACB54931 sequence only revealed three different amino acids, showing the high identity of the sequence and predicted structure (Supplementary Figures S2, S3).

**FIGURE 2:**
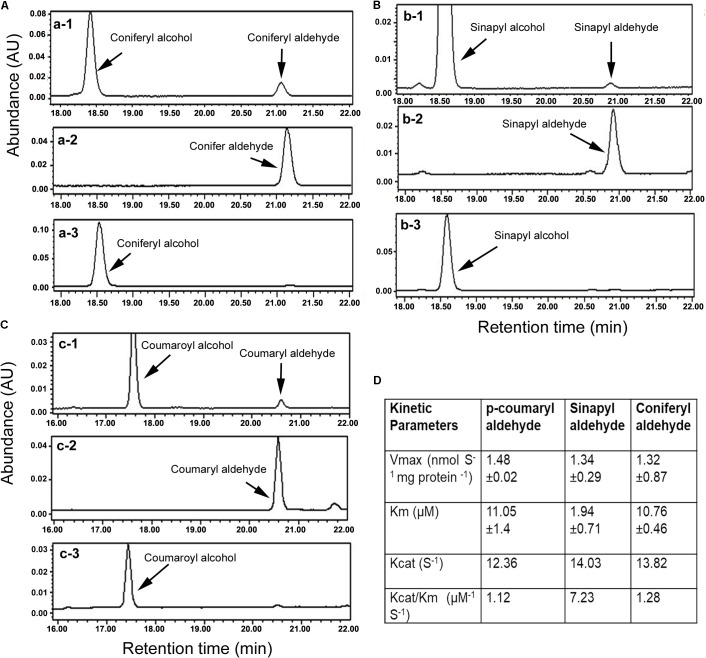
Characterization of enzymatic activity with HPLC analysis and kinetic features. **(A)** HPLC profiles show conversion of coniferyl aldehyde to coniferyl alcohol, a-1: reaction product and substrate, a-2: coniferyl aldehyde standard, and a-3: coniferyl alcohol standard. **(B)** Profiles of HPLC show conversion of sinapyl aldehyde to sinapyl alcohol, b-1: reaction product and substrate, b-2: sinapyl aldehyde standard, and b-3: sinapyl alcohol standard. **(C)** Profiles of HPLC show conversion of p-coumaryl aldehyde to p-coumaryl alcohol, c-1: reaction product and substrate, c-2: p-coumaryl aldehyde standard, and c-3: p-coumaryl alcohol standard. **(D)** Km, Vmax, Kcat, and Vcat/Km values were calculated for three substrates. All experiments in **A–C** were repeated at least three times. All experiments in **D** were carried out with three replicates to calculate the values.

The ORF was cloned into pDEST17 (6xHis tag) to induce the recombinant AaCAD. The purified recombinant enzyme was further obtained using Ni-NTA Superflow Columns (Supplementary Figure S1). Three substrates, coniferyl aldehyde, sinapyl aldehyde, and p-coumaryl aldehyde, were used to evaluate the enzymatic activity. After the recombinant AaCAD was incubated with the three substrates, HPLC analysis showed that the recombinant enzyme efficiently converted the three substrates to their corresponding alcohol products, coniferyl alcohol, sinapyl alcohol, and p-coumaryl alcohol, respectively (**Figures 2A–C**). These results demonstrated the biochemical involvement of AaCAD in the lignin biosynthetic pathway (**Figure 1**).

Kinetic analysis was carried out to characterize Km, Vmax, Kcat, and Kcat/Km values for the three substrates. Given that coniferyl alcohol, sinapyl alcohol, and p-coumaryl alcohol produced from AaCAD catalysis have maximum absorbance values at 340 nm, the use of a microplate reader is a highly efficient way to measure their concentrations in the same reaction time frame. The resulting data showed that the Km value of sinapyl aldehyde was lower than those of coniferyl aldehyde and p-coumaryl aldehyde, which had similar values (**Figure 2D**). This result supports a catalytic preference for sinapyl aldehyde. The Vmax values for the three substrates were similar. The Kcat/Km value for sinapyl aldehyde was higher than those of coniferyl aldehyde and p-coumaryl aldehyde, which had similar values (**Figure 2D**).

### Overexpression of *AaCAD* in SP *A. annua*

**FIGURE 3:**
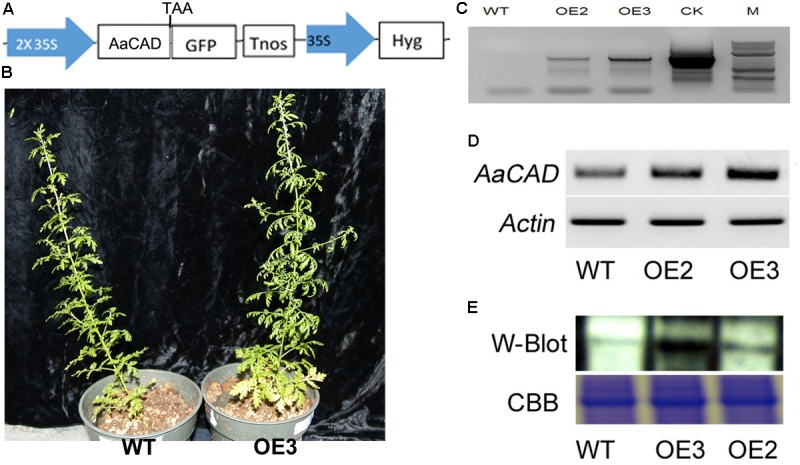
A simplified map of the T-DNA cassette and integration of the transgene in transgenic plants. **(A)** A T-DNA cassette constructed in a binary vector showing the use of the 2 × 35S promoter for overexpression of *AaCAD* in *A. annua*. **(B)** Images show phenotypes of an OE3 transgenic line vs. one wild-type control plant. **(C)** PCR gel images show the genomic DNA fragment amplified from two transgenic lines using a pair of primers consisting of a forward 35S promoter primer and a reverse CAD primer. **(D)** Gel images show the increased expression of *AaCAD* in transgenic plants. **(E)** Gel images show the increased AaCAD protein in transgenic lines. Abbreviations in T-DNA: AaCAD, *Artemisia annua* cinnamyl alcohol dehydrogenase; GFP, green florescent protein; Hyg, hygromycin; Tnos, Nos terminator. Plant name abbreviation: OE2 and OE3, two transgenic lines; WT, wild-type control. PCR and RT-PCR experiments were performed with three biological replicates, each with at least three replicates. Western blotting was repeated three times.

The ORF of *AaCAD* was cloned into the PMDC-84 vector, and its expression was driven by a 2 × 35S promoter (**Figure 3A**). This construct was introduced into SP *A. annua* via *A. tumefaciens*-mediated transformation of leaf explants. Numerous hygromycin-resistant shoots were regenerated from infected explants and further rooted to obtain multiple transgenic plantlets. More than 10 plantlets were planted in soil contained in pots and grown in the phytotron as reported previously (Ma et al., 2017a). As shown in the photograph of line OE3 and a wild-type plant in **Figure 3B**, the transgenic candidates grew similarly. In addition, vector control transgenic plants such as *ADS* transgenic plants (Ma et al., 2015) were used as a vector control, and no difference in plant growth was observed between *ADS* and AaCAD transgenic plants.

Based on the 35S promoter and *AaCAD* sequences, primers were designed for PCR-based genotyping. The resulting PCR data, as shown for transgenic examples OE2 and OE3 in **Figure 3C**, demonstrated the integration of the 35S and *AaCAD* transgenes into the genome of transgenic candidates. These two candidate lines were further selected for RT-PCR and western blot analysis. RT-PCR using gene-specific primers showed higher expression levels of *AaCAD* in these two transgenic plants compared with the wild-type control (**Figure 3D**). Western blot analysis demonstrated an increased protein level of AaCAD in the two transgenic plants (**Figure 3E**). These data demonstrate that the two candidates are transgenic plants.

**FIGURE 4:**
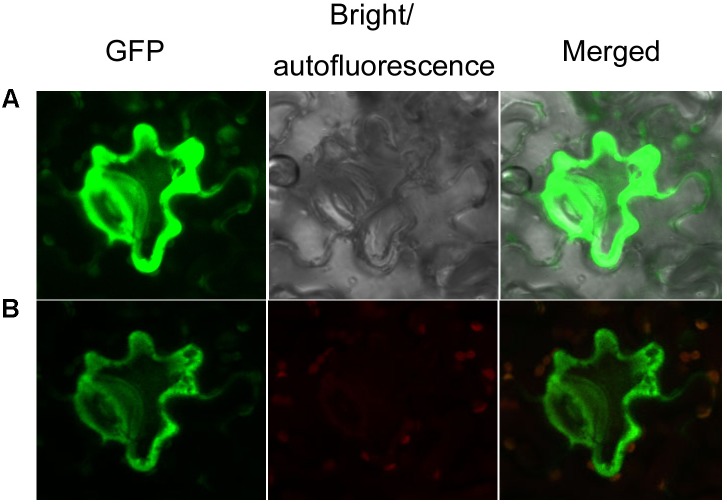
Subcellular localization of AaCAD-EGFP. **(A)** The green fluorescence signal of GFP in cells is shown in images photographed using green and bright channels. **(B)** The green fluorescence signal of GFP in cells is shown in images photographed using green and red channels. Green, green fluorescence of GFP; red, autofluorescence of chloroplasts; merged, green fluorescence of GFP and red autofluorescence of chloroplasts. This experiment was repeated three times, each with at least three biological replicates.

To characterize the subcellular localization of AaCAD, confocal microscopy analysis was performed. The stop codon TAA was removed from the *AaCAD* ORF. The resulting TAA-eliminated sequence was fused to the N-end of an EGFP for protein localization. As reported previously for testing transgene functions in SP *A. annua* (Ma et al., 2017a,b), transient expression of AaCAD-EGFP was carried out in *Nicotiana benthamiana*. Both green and red channels were used to localize the proteins. The resulting data from two-channel observations showed that epidermal cells exhibited strong green fluorescence in the cytosol (**Figure 4**), indicating that AaCAD catalyzes reactions in the cytosol.

### Lignin and Coumarin Contents Are Increased in Transgenic Plants

**FIGURE 5:**
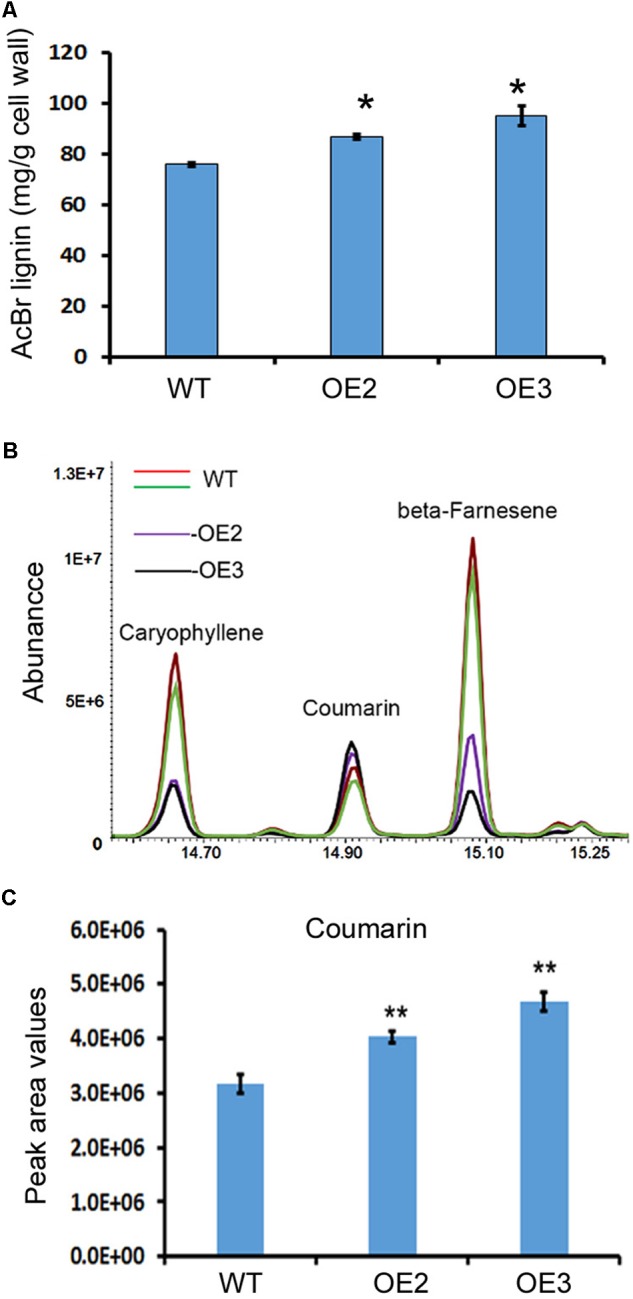
Overexpression of *AaCAD* increases the lignin and coumarin contents in transgenic plants. **(A)** AcBr analysis-based calculation showing the lignin content in leaves of two transgenic lines and wild-type plants. **(B)** Overlay of total ion chromatographs showing increases in coumarin abundance in leaves of the two transgenic lines. **(C)** Peak area values show significant increases in coumarin in leaves of the two transgenic lines. OE2 and OE3, two transgenic lines overexpressing the *AaCAD* transgene; WT, wild-type. ^∗^ in **(A)** and ^∗∗^ in **(C)** denotes significant differences between transgenic lines and wild-type plants (*N* = 3, *p* < 0.01). All analyses were conducted with three biological replicates, each repeated three times.

Lignin and other phenylpropanoid metabolites were analyzed in the transgenic and wild-type plants. Given that T0 transgenic plants were regenerated at different time points, in this study, we mainly focused on OE2 and OE3, which were regenerated at the same time and grew in a synchronous manner. The main stem and all branches of OE2 and OE3 were harvested for lignin analysis after the seeds were collected. Acetyl bromide (AcBr) analysis was completed to measure the lignin contents. The resulting data showed that lignin was significantly increased in the stems and branches of these two transgenic lines (**Figure 5A**).

Coumarin (2H-chromen-2-one) was annotated by GC-MS analysis. The resulting data showed a significant increase in coumarin content in the leaves of these two lines compared with the leaves of the wild-type plants (**Figures 5B,C**), demonstrating that AaCAD is associated with coumarin formation in plants.

### Artemisinin, Arteannuin B, and Other Sesquiterpenes Are Decreased in Transgenic Plants

**FIGURE 6:**
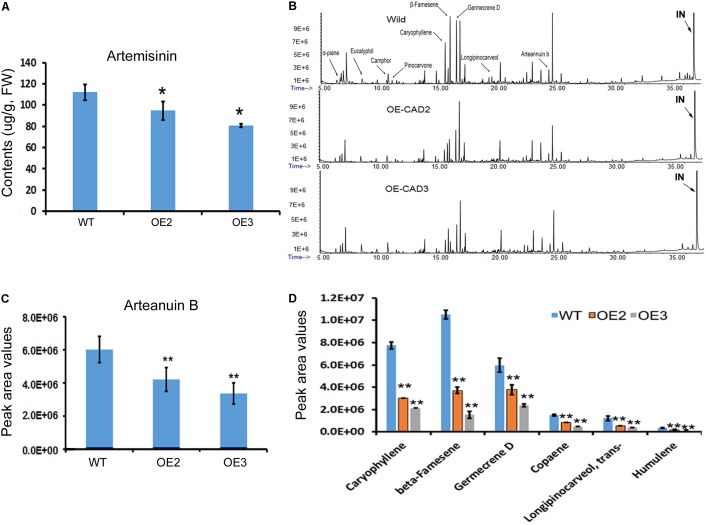
Effects of *AaCAD* overexpression on levels of artemisinin, arteannuin B, and other sesquiterpenes in leaves of transgenic plants. **(A)** LC-MS analysis measurements show decreases in artemisinin contents. **(B)** Overlay of total ion chromatographs shows the abundance reduction of numerous non-polar metabolites, among which arteannuin B and sesquiterpenes are labeled as follows: “IN”, internal metabolite as a control. **(C)** Peak area values show the significant reduction of arteannuin B. **(D)** Peak area values show the reduction of five sesquiterpenes. ^∗^ in **(A)** and ^∗∗^ in **(C)** and **(D)** denote a significant difference in the transgenic lines compared with WT plants. (*N* = 3, *p* < 0.01). All experiments were carried out with three biological replicates, each with three replicates.

Artemisinin, arteannuin B, and other sesquiterpenes were profiled in the leaves of *AaCAD* transgenic vs. wild-type plants. As reported previously (Ma et al., 2015), artemisinin was measured using HPLC-MS. The resulting data showed that the contents of artemisinin were reduced significantly in two transgenic lines compared with wild-type plants (**Figure 6A**). In addition, arteannuin B and other sesquiterpenes were analyzed using GC-MS as reported previously (Ma et al., 2015). The resulting total ion chromatographs showed that the abundance of many non-polar metabolites was reduced in the two transgenic lines compared with wild-type plants (**Figure 6B**). Peak deconvolution allowed annotation of arteannuin B and other sesquiterpenes (**Figure 6B**). Peak values were recorded for arteannuin B and six other main sesquiterpenes. The resulting data showed that the peak values of arteannuin B and six sesquiterpenes were significantly reduced in the transgenic plants compared with wild-type controls (**Figures 6C,D**). These data indicate that the biosynthetic activity of sesquiterpenes is reduced in *AaCAD* transgenic plants. To determine whether the reduction of these metabolites was associated with the density of glandular trichomes, leaves and stems of transgenic vs. wild-type plants were examined using a Leica MZ FLIII fluorescence stereomicroscope and photographed (Supplementary Figures S4, S5). The resulting data showed that although many transparent or semi-transparent trichomes were observed in both abaxial and adaxial surfaces, most of them are T-shaped trichomes (Supplementary Figure S5) or other sharp stick trichomes. The density of glandular trichomes was low and similar on those leaves between transgenic and wild-type plants (Supplementary Figure S4).

## Discussion

The present study shows that understanding the function of AaCAD, a short chain oxidoreductase in *A. annua*, is fundamental for the metabolic engineering of this medicinal crop to achieve high production levels of artemisinin. Numerous studies have reported that CAD is a key enzyme in the biosynthesis of lignin and plays an essential role in plant development associated with plant biomass (Mitchell et al., 1994; Feuillet et al., 1995; Halpin et al., 1998; Bagniewska-Zadworna et al., 2014; Pan et al., 2014; Choi et al., 2016). Downregulation of *CAD* expression or its knockout causes a reduction of lignin, which leads to severe plant dwarfism and a decreased biomass (Sirisha et al., 2012; Anderson et al., 2015; Ozparpucu et al., 2017; Ponniah et al., 2017) as well as decreased plant resistance to pathogens (Bagniewska-Zadworna et al., 2014; Preisner et al., 2014; Rong et al., 2016). However, whether an increase in total lignin via *CAD* overexpression can affect the biosynthesis of terpenoids, such as artemisinin biosynthesis in *A. annua*, remains uninvestigated. We recently reported the cloning of a *CAD* homolog from a cross-pollinating heterozygous cultivar and its biochemical analysis. Our previous *in vitro* enzyme assays showed that the recombinant CAD used coumaryl, coniferyl, and sinapyl aldehydes as substrates in the presence of NADPH (Li et al., 2012). Our previous enzyme assays also showed that this CAD could use artemisinic aldehyde, a sesquiterpenoid metabolite, as a substrate to produce to artemisinic alcohol. Although the Km value of CAD for artemisinic aldehyde is higher than those of coumaryl, coniferyl, and sinapyl aldehydes, the activity using this sesquiterpenoid substrate indicates its catalytic promiscuity. Accordingly, we hypothesize that CAD not only essentially involves lignin formation, but it can also control artemisinin accumulation in *A. annua*. However, a functional analysis of CAD *in planta* has not been performed, given that the cross-pollinating cultivar used to clone *CAD* is a heterozygous species that causes progeny segregation. The heterozygous cultivar is not appropriate material to generate transgenic plants to understand this gene function *in planta*. Herein, we re-cloned a new homolog from our novel inbred SP *A. annua* (F3 progeny) and designated it *AaCAD* to further characterize its functions *in vitro* and *in vivo*. Our enzyme assay showed that this new recombinant AaCAD efficiently converted coumaryl, coniferyl, and sinapyl aldehydes to their corresponding alcohols (Supplementary Figure S2). As expected, the overexpression of *AaCAD* significantly increased the lignin content in SP *A. annua* plants (**Figure 5A**), demonstrating its involvement in lignin biosynthesis. In addition, overexpression increased the content of coumarin (**Figures 5B–C**), indicating its additional function in the phenylpropanoid pathway. Given that amino acid sequence analysis showed that this AaCAD and the previous one from a cross pollinating cultivar had only three amino acid differences (Supplementary Figure S2) and structural modeling showed the same structure conformation (Supplementary Figure S3), two homologs were anticipated to have similar enzymatic activity to convert artemisinic aldehyde to alcohol. As anticipated, the overexpression of this new *AaCAD* led to significant decreases in the contents of artemisinin, arteannuin B, and other sesquiterpenes in leaves (**Figures 6A,C**). Although these results were not a goal of our metabolic engineering strategy, they supported the findings of our previous *in vitro* enzyme assay showing that recombinant CAD from a cross-pollinating *A. annua* cultivar converted artemisinic aldehyde to artemisinic alcohol (Li et al., 2012), a late step in the reverse direction of the artemisinin pathway (**Figure 1**). To understand whether this reaction could occur in the cytosol of transgenic plant cells, we used GFP fusion to characterize the subcellular localization. Our confocal microscopy analysis revealed the cytosolic localization of AaCAD (**Figure 4**). These data further revealed that the cytosolic localization of AaCAD could reverse the step toward the formation of artemisinin catalyzed by CYP71AV1, CPR1, and AaADH1 and thus catalytically reduce the efficacy of three enzymes in the cytosol. These data indicate that overexpression of *AaCAD* negatively controls the formation of artemisinin in *A. annua*.

Glandular trichomes are the localization of the artemisinin biosynthesis (Duke et al., 1994; Xiao et al., 2016). A field study reported that application of salicylic acid or chitosan oligosaccharidea (out of different stress treatments) could significantly decrease 4–9 glandular trichomes per square of millimeter only on upper leaves from mature plants grown in the field (Kjaer et al., 2012). In the same study, three plants selected from the field were propagated via cutting and the resulting clones were grown in the greenhouse. Kjaer et al. (2012) observed that application of nine stress treatments did not affect the density of glandular trichomes on upper leaves but two stress conditions could significantly reduce about two trichomes per square of millimeter on lower leaves of clones. These results indicate that the density of glandular trichomes can be affected by external stress conditions. In our study, although whether *CAD* can alter glandular and other trichome development remains unknown, we examine glandular trichomes on leaves from 8–12 nodes and those nodes. We did not observe significant density difference of glandular trichomes between wild-type and transgenic plants. It was interesting that certain visual alterations in T-shaped trichomes were observed under microscope (Supplementary Figure S4). Given that glandular trichomes are the tissue of the artemisinin biosynthesis, our herein observation indicates a valuable interest in future studies to thoroughly examine glandular trichomes on all leaves and their effects on artemisinin contents in *CAD* transgenic progeny.

The increase in lignin and the reduction of artemisinin and other sesquiterpenes reveal an interesting crosstalk between two completely distinct pathways via AaCAD catalysis in transgenic plants. Lignin biosynthesis is mainly specialized in vascular tissues in all land plants (Weng and Chapple, 2010; Espineira et al., 2011). By contrast, artemisinin biosynthesis is mainly observed in *A. annua* and potentially other *Artemisia* species. Furthermore, this unique pathway is considered to be limited to a type of specialized glandular trichomes on leaves and flowers (Covello et al., 2007), although its cellular specialization remains controversial (Xie et al., 2016). This type of crosstalk in transgenic plants is most likely associated with two pathway intermediates and co-localization in the cytosol, despite their metabolic distinction (**Figure 1**). In the present transgenic plants, the *AaCAD* transgene was driven by two 35S promoters (**Figure 3A**), leading to constitutive overexpression of the transgene in the tissues, including trichomes. Our previous *in vitro* assay also showed that the recombinant AaCAD catalyzed the conversion of artemisinic aldehyde to alcohol (Li et al., 2012) (**Figure 1**). Accordingly, it is likely that overexpression of AaCAD (**Figure 3E**) enhances the catalytic conversion of artemisinin aldehyde to alcohol in the presence of NADPH in trichomes. In addition to our observations, CAD or CAD-like enzymes have been reported to be involved in the formation of monoterpenoid indole alkaloids in *Rauvolfia serpentine* (Geissler et al., 2016). Moreover, additional biochemical studies have shown that CAD is characterized by a high promiscuity level of different substrates (Chao et al., 2014; Geissler et al., 2016). Based on our and other reports, one hypothesis is that CAD-mediated crosstalk can occur in other metabolic pathways. As more studies are performed, it is anticipated that this type of enzyme-based crosstalk will be observed in different plants. In summary, the observed AaCAD-mediated crosstalk leading to reduced artemisinin contents is instructional for the engineering of *A. annua* for high production.

In conclusion, a new *AaCAD* was cloned from and overexpressed in inbred SP *A. annua*. The enzyme is localized in the cytosol. Overexpression of *AaCAD* in *A. annua* increased lignin and coumarin contents, but it decreased artemisinin, arteannuin B, and other sesquiterpene contents. These data revealed an AaCAD-mediated metabolic crosstalk between the phenylpropanoid (lignin and coumarin) and the sesquiterpene (artemisinin) pathways in the cytosol.

## Author Contributions

DM and D-YX conceived and designed the research. DM, CX, FA-G, JY, HW, RJ, and D-YX performed the experiments and analyzed the data. DM and D-YX wrote the manuscript. D-YX supervised the entire project. All authors read and approved the manuscript.

## Conflict of Interest Statement

The authors declare that the research was conducted in the absence of any commercial or financial relationships that could be construed as a potential conflict of interest.

## Footnotes

1 http://www.ebi.ac.uk/Tools/msa/clustalo/
