# Supplementary material for: Overexpression of Artemisia annua Cinnamyl Alcohol Dehydrogenase Increases Lignin and Coumarin and Reduces Artemisinin and Other Sesquiterpenes
Source: Front Plant Sci. 2018 Jun 19;9:828. doi: 10.3389/fpls.2018.00828 (PMC6018409; doi:10.3389/fpls.2018.00828)
Supplement: Supplementary file 1 [file Data_Sheet_1.PDF]

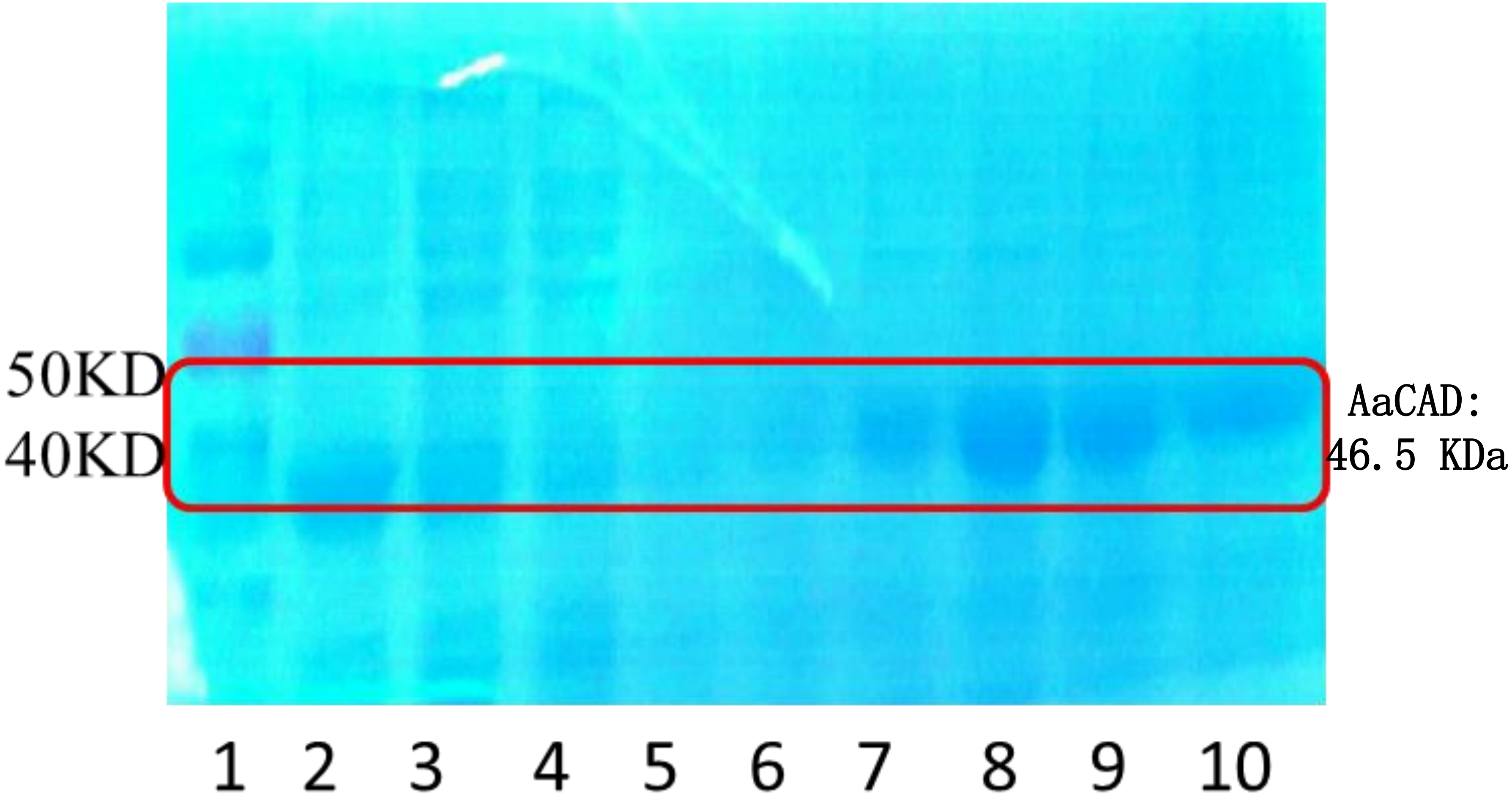

Supplementary Fig. 1 SDS-PAGE image showing purified soluble recombinant AaCAD. 1: protein ladder; 2: total protein; 3: total soluble crude protein; 4: flow-through protein; 5: protein collection after washing buffer; 6-10: AaCAD eluate from column step 5.

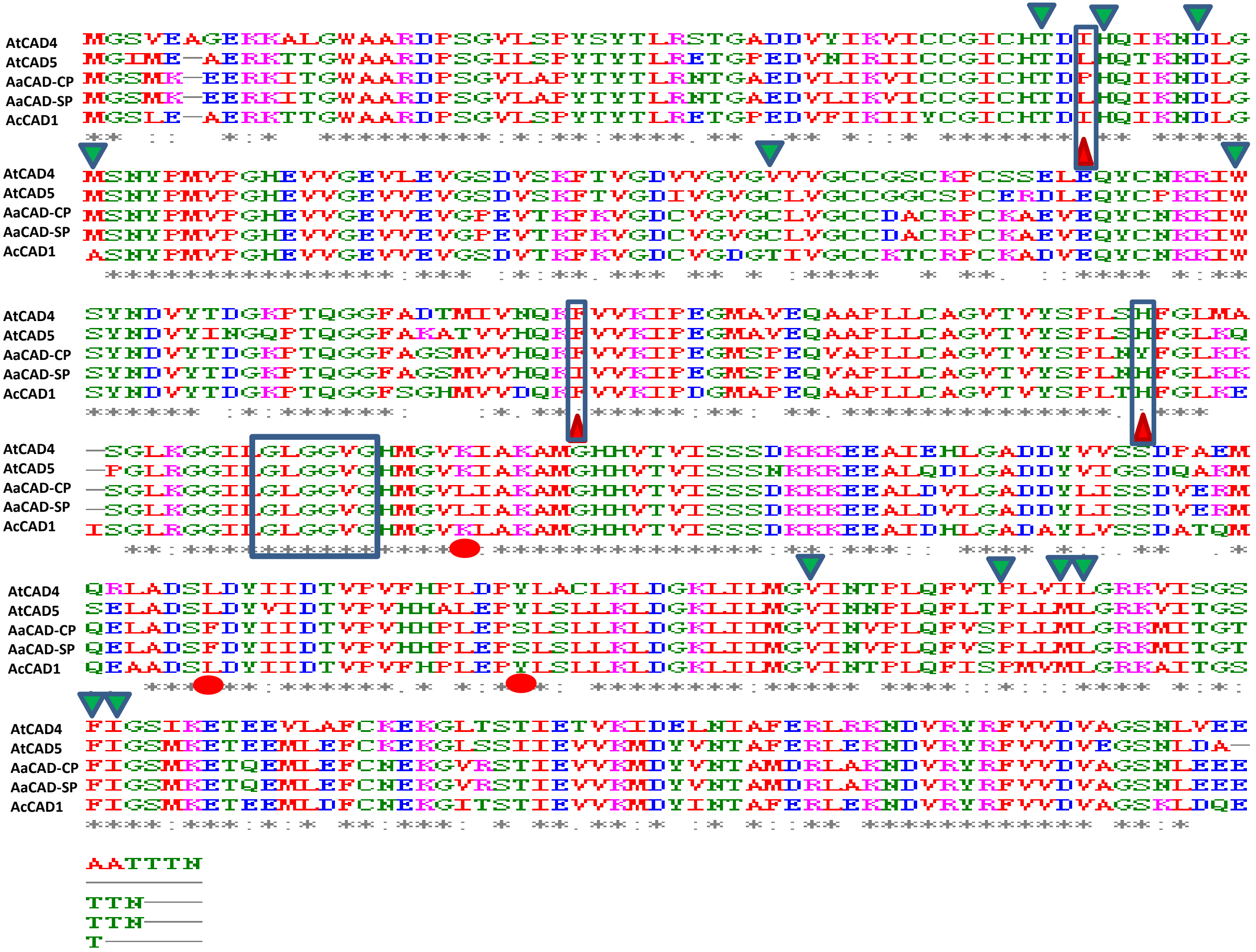

Supplementary Fig. 2 Alignment of the amino acids of two *Artemisia annua* cinnamyl alcohol dehydrogenases from self-pollination (AaCAD-SP) and cross-pollution (AaCAD-CP) cultivars (Li et al. 2012) using the online Cluster Omega program (http://www.ebi.ac.uk/Tools/msa/clustalo/). Residues in the box indicate a highly conserved 188 GLGGVG193 motif in CAD. The putative substrate-binding pocket residues are marked with filled green triangles. The three red triangles represent different residues between AaCAD-SP and AaCAD-CP. Red dots represent residues that are conserved in all CADs, but they do not appear in two AaCADs.

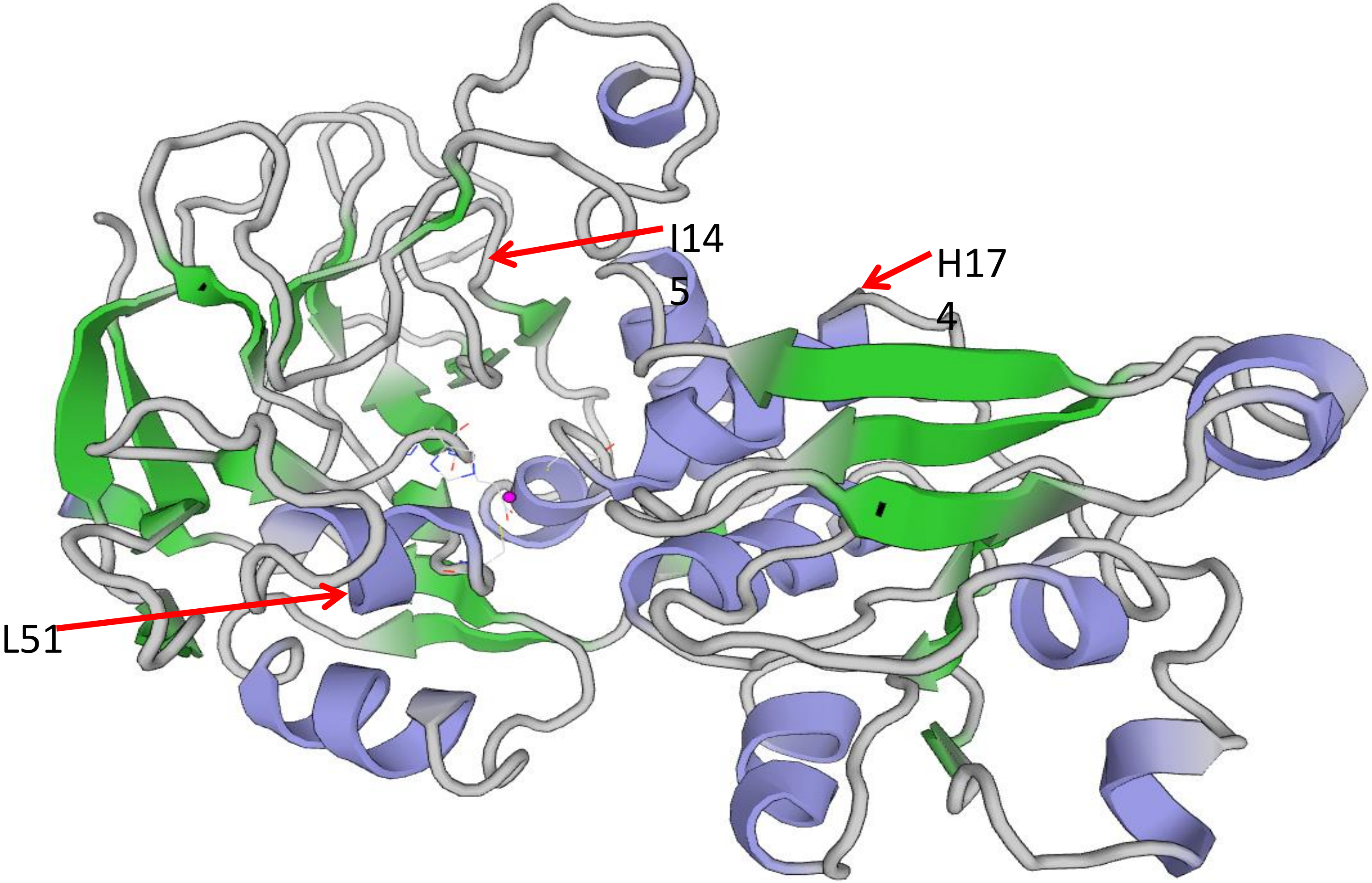

Supplementary Fig. 3 The amino acid sequence of AaCAD from SP *A. annua* was uploaded into the SWISS-MODEL server (https://www.swissmodel.expasy.org). L51, I 145 and H174 are three different amino acids in AaCAD homologs from SP and CP *A. annua*.

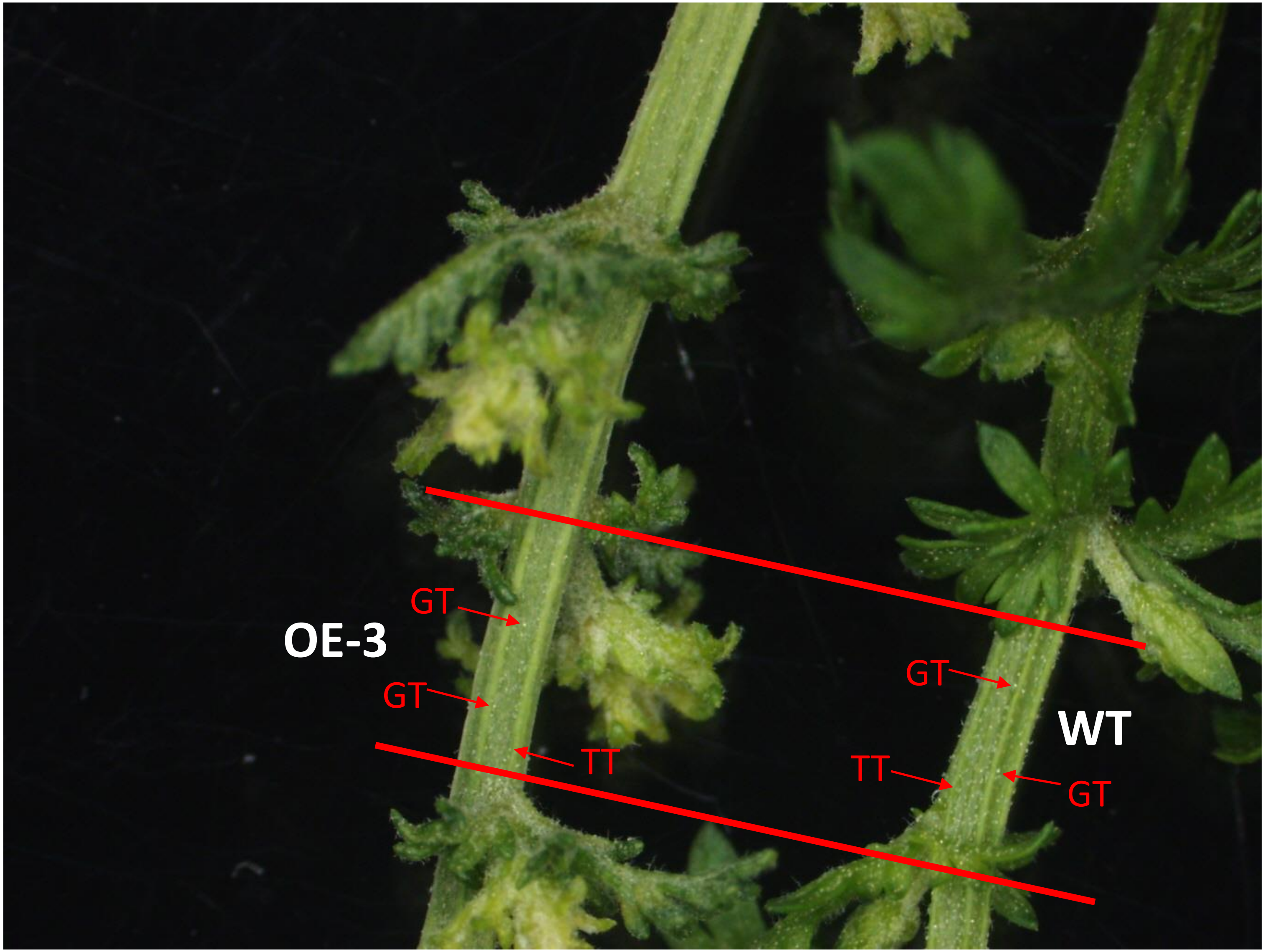

Supplementary Fig. 4 An image of leaves and stems collected from 8-12 nodes of two-month-old plants grown in the phytotron. The density of glandular trichomes was similar between the transgenic (OE3) and wild type plants. Images were photographed using a Leica MZ FLIII fluorescence stereomicroscope with an Olympus DP71 digital camera. GT: glandular trichomes, TT: T-shaped trichomes.

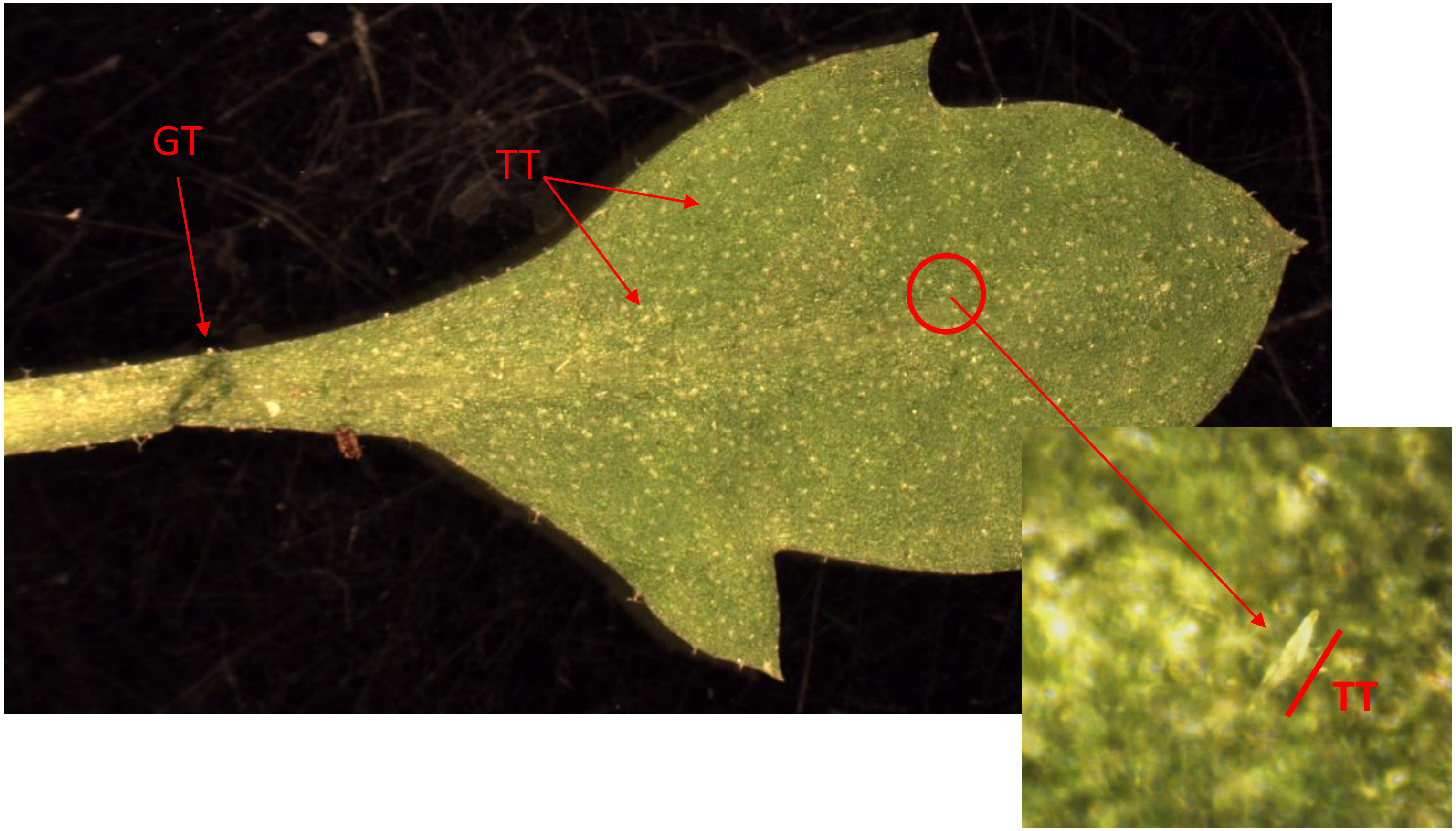

Supplementary Fig. 5 An image showing the abaxial surface of a leaflet section. Many dot structures with strong lighting reflection were observed from the surface of leaftlets and indented sections. From the top view, they look glandular structures. However, examination of those dots (from 20 leaves) under stereo microscope shows that most (approximately 98%) of them are a T-shaped structure or sharp stick structure. GT: glandular trichomes, TT: T-shaped trichomes.
